# Should adrenal incidentaloma patients be evaluated for muscle mass, function, and quality? A cross-sectional study

**DOI:** 10.1007/s12020-025-04170-6

**Published:** 2025-01-26

**Authors:** Samet Alkan, Sedat Can Guney, Can Akcura, Nilufer Ozdemir, Zeliha Hekimsoy

**Affiliations:** Department of Endocrinology and Metabolic Diseases, Manisa Celal Bayar University Hospital, Manisa, Turkey

**Keywords:** Adrenal incidentaloma, Mild autonomous cortisol secretion, Sarcopenia, Skeletal muscle mass, EWGSOP2

## Abstract

**Purpose:**

Our study evaluated skeletal muscle mass, function and quality among mild autonomous cortisol secretion (MACS) patients and non-functioning adrenal incidentaloma (NFAI) patients in comparison with the control group without adrenal mass.

**Methods:**

63 NFAI (49 female, 14 male) and 31 MACS (24 female, 7 male) patients were included in the study. As the control group, 44 patients (31 women, 13 men) who were known to have no radiological adrenal pathology on computed tomography or magnetic resonance imaging performed for other reasons were selected. After recording the laboratory parameters of the patients, anthropometric measurements, handgrip strength test with dynamometer, SARC-F survey and bioelectrical impedance analysis (BIA) measurements were performed.

**Results:**

There was no statistical difference among the groups in terms of age, gender, and BMI parameters. Handgrip strength (HGS), skeletal muscle mass (SMM) index (SMM/BMI), and skeletal muscle quality (HGS/SMM), values used to evaluate muscle strength and quality, were found to be significantly lower in both the MACS and NFAI groups compared to the control group (*p* = 0.004, *p* = 0.012 and *p* = 0.034 respectively). This significance was also present in women subgroup analyses (*p* = 0.002, *p* = 0.037 and *p* = 0.039 respectively), but these parameters lost their statistical significance in men. In the correlation analysis of the female subgroup, 24-h free urine cortisol value was inversely proportional to skeletal muscle quality (r_s_ = -0.417, *p* = 0.008).

**Conclusion:**

Our study showed that there is a decrease in muscle mass and function in female AI patients, and this decrease is more severe in MACS patients. These results may suggest that mild cortisol excess also has negative effects on skeletal muscle metabolism.

## Introduction

Sarcopenia is characterized by a decrease in muscle mass and loss of muscle functions and quality. This condition causes deterioration in mobility, decrease in physical endurance and walking pace, physical limitation and increases mortality and morbidity [1]. Global working groups such as the European Working Group on Sarcopenia in Older People (EWGSOP2), Asian Working Group for Sarcopenia (AWGS), Foundation for the National Institutes of Health Sarcopenia Project (FNIH) and International Working Group on Sarcopenia (IWGS) contributed to the operational definition of sarcopenia and standardization of studies. In the past, sarcopenia was mainly associated with aging, but nowadays, it is accepted that the development of sarcopenia begins earlier in life and there are many other underlying reasons [2].

Adrenal incidentaloma (AI) is a term used for adrenal masses detected incidentally when imaging is performed for another indication, without any obvious clinical features of adrenal disease. The frequency of AI is constantly increasing with the widespread use of medical imaging techniques. The prevalence increases with aging and varies according to radiological studies and autopsy series. While it affects approximately 2% of the general population, this rate increases to about 7% in individuals over the age of 70 [3]. The majority of adrenal incidentalomas (about 40–70%) consist of non-functional adrenal incidentalomas (NFAI) in which hormone hyperfunction cannot be detected through clinical and hormonal evaluation. Mild hypercortisolemia, the detection of elevated cortisol levels without apparent clinical evidence, is the most common hormonal disorder (about 20–50%) in adrenal incidentaloma cases. The authors of the 2023 European Society of Endocrinology (ESE)-European Network for the Study of Adrenal Tumors (ENSAT) recommend using the term “mild autonomous cortisol secretion” (MACS) for this clinical condition [4]. Studies have revealed that MACS (formerly known as subclinical Cushing’s syndrome) is associated with metabolic and cardiovascular complications such as hypertension, type 2 diabetes mellitus (DM), metabolic syndrome, obesity and osteoporosis [5].

Loss of muscle mass and function caused by increased sustained cortisol release is typical in Cushing’s syndrome (CS) [6, 7]. Proximal muscle weakness is a common symptom among CS patients [8]. Glucocorticoids, including cortisol, have catabolic effects on many tissues, including muscle tissue. Glucocorticoids cause protein degradation in skeletal muscle by inducing atrogin-1 and MuRF-1 in the ubiquitin-proteasome system [9]. Cortisol also inhibits the production of IGF-1, an anabolic hormone. Additionally, cortisol increases myostatin production from myocytes; this inhibits myogenesis (muscle cell growth and differentiation) by affecting the autocrine function of muscle cells [10]. Although the effects of overt hypercortisolemia caused by CS on muscle mass and function are known, studies on muscle mass, function and quality of MACS are limited. Our study evaluated skeletal muscle mass, function and quality among MACS and NFAI patients in comparison with the control group without adrenal mass.

## Materials and methods

### Study participants and protocol

This study was performed between February 2023 and February 2024 at Manisa Celal Bayar University Endocrinology and Metabolism clinic. 162 patients admitted due to adrenal mass were evaluated.

Patients’ sociodemographic data such as smoking habit, alcohol intake ( ≥3 units/day), exercise status, history of medication, previous surgical procedures, and menstrual status in women were recorded using an interviewer-assisted questionnaire.

### Radiological evaluation

All patients underwent a computed tomography (CT) scan via a 256 detector CT scanner (SOMATOM Drive, Siemenes Healthineers, Germany) with the following imaging parameters: maximum scan speed 458 mm/sec, spatial resolution 0.30 mm, slice thickness 1 mm, 1:1 table pitch, 70–140 kV @ 10 kV steps, variable tube current depending on patient size (650 mA, 750 mA). AI diagnosis was confirmed upon the detection of an adrenal lesion bigger than 1 cm in diameter and with an appearance compatible with adenoma. 10 patients were excluded due to benign non-adenoma lesions (e.g. adrenal myelolipoma). The size, localization (right, left, bilateral) and Hounsfield unit (HU) of the lesions were recorded. In the presence of more than one adenoma on one gland, the larger one was taken into account.

### Laboratory tests

All patients underwent 1-mg overnight dexamethasone suppression test (1-mg DST) and 24-h urinary fractionated metanephrines measurement. Cortisol was measured by electrochemiluminescence immunoassay method using a UniCel DxI 800 (Beckman Coulter, Inc.) device. Urinary fractionated metanephrines were measured by liquid chromatography mass spectrometry (LC-MS/MS) at an external laboratory (Agilent 6460 Triple Quadropol). We excluded patients with high urinary fractional metanephrine levels and clinical findings consistent with pheochromocytoma. If the patient had hypertension and/or hypokalemia, plasma aldosterone and plasma renin activity levels were additionally measured and their ratio was calculated (PA/PRA). Plasma aldosterone and plasma renin activity was measured by the electrochemiluminescence immunoassay method using the LIAISON® XL device (DiaSorin S.p.A.). Patients with a PA/PRA ratio above 20 were referred for confirmatory tests for hyperaldosteronism and were excluded. Patients with a cortisol level of >1.8 μg/dL after the 1-mg DST were admitted to the inpatient clinic for further examination. After 2 days of hospitalization, basal ACTH and cortsiol levels, dehydroepiandrosterone sulfate (DHEA-S), 24-h urinary free cortisol (UFC) and midnight cortisol were measured. Free urinary cortisol was measured by electrochemiluminescence immunoassay method using a UniCel DxI 800, (Beckman Coulter, Inc.) device (reference range: 20.4–292.3 µg/ 24 h). DHEA-S was measured by the electrochemiluminescence immunoassay method using ADVIA Centaur® XPT (Siemens Healthineers AG) device (reference range: Female: 35–430 μg/dL and Male: 80–560 μg/dL). ACTH was measured by the electrochemiluminescence immunoassay method using the Immulite 2000 (Siemens Healthineers AG) device (reference range: 0-46 pg/mL). 1-mg DST was repeated and patients with a cortisol level of >1.8 μg/dL underwent a two-day, 2 mg test. We accepted the patients without signs and symptoms of overt CS and post dexamethasone serum cortisol concentration above 1.8 µg/dL (both 1-mg DST and two-day, 2 mg test) as MACS regardless of cortisol value. Also, we confirmed ACTH-independence in these patients. We used the post 1-mg DST cortisol value ≤ 1.8 μg/dL criterion for the diagnosis of NFAI. Finally, we classified 63 patients as NFAI (49 women and 14 men) and 31 patients as MACS (24 women and 7 men).

### Exclusion criteria

We excluded 58 more subjects who were suspected to have adrenal CS (n = 3), primary hyperaldosteronism (n = 8), pheochromocytoma (n = 2), adrenal carcinoma (n = 1), adrenal metastasis (n = 2), other organ malignancies (n = 9), diabetic patients under insulin treatment or have poorly controlled diabetes (Hba1c >%9) (n = 11), patients with cardiac pacemakers or metal bone prostheses (n = 6), a history of drugs that affect muscle tissue (such as steroid, estrogen, testosterone) (n = 3), overt hyperthyroidism or hypothyroidism (n = 6) and patients with organ failure (chronic renal failure, liver cirrhosis, heart failure with reduced ejection fraction) (n = 7).

### Control group

**Fig. 1:**
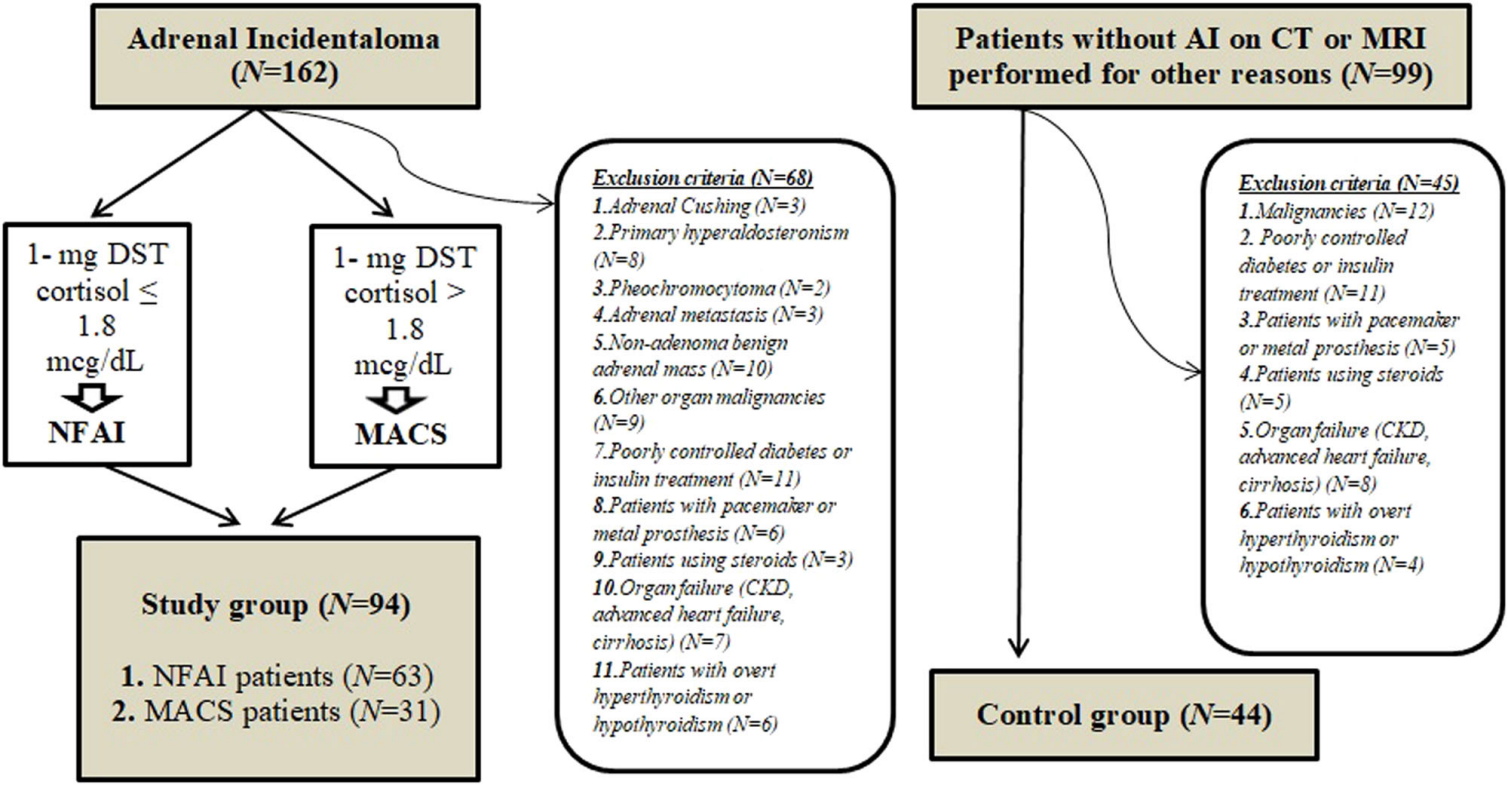
Study and control group flow diagram and exclusion criteria * DST dexamethasone suppression test, NFAI non-functional adrenal incidentaloma, MACS mild autonomic cortisol secretion, AI adrenal incidentaloma, CT computed tomography, MRI magnetic resonance imaging, CKD chronic renal failure

As the control group, 44 patients (31 women, 13 men) who were admitted to our outpatient clinic on the same time interval and who were known to have no adrenal pathology on computed tomography or magnetic resonance imaging performed for other reasons (e.g. investigation of the etiology of abdominal pain, COVID-19 infection) were included. The study group flow diagram and exclusion criteria are shown in Fig. 1.

### Measuring sarcopenia parameters

The European Working Group on Sarcopenia in the Elderly 2018 (EWGSOP-2) consensus was accepted as a guide for the evaluation of sarcopenia parameters [11].

### Anthropometric measurements

The patients’ height, weight, body mass index (BMI), waist circumference, mid-arm circumference, and calf circumference were measured with a non-elastic but flexible plastic tape by the same researcher. BMI was obtained by dividing body weight (kg) by height squared (m^2^). Waist circumference was measured from the midpoint between the lowest rib and the iliac crest. Mid-arm circumference was measured at the middle point between the acromion and the olecranon process while elevating and internally rotating the arm. Calf circumference was measured at the widest calf circumference level with participants standing.

### Assessing the risk of sarcopenia

To evaluate the risk of sarcopenia, the Turkish validated form of the SARC-F questionnaire was used [12]. The questionnaire consists of 5 questions each worth a maximum of two points and a score of 4 or above is considered as a risk for sarcopenia [13].

### Muscle strength

The hand grip strength (HGS) was used to evaluate muscle strength. HGS was assessed with a Baseline® hydraulic hand dynamometer (Fabrication Enterprises, Inc., White Plains, NY, USA). All measurements were performed three times in the morning in a sitting position, with the elbow at 90° flexion and the wrist in the neutral position with both hand by the same researcher. 30 s of resting was allowed between each measurement [14]. The maximum measurement value was recorded (unit in kilogram) as the HGS.

### Muscle quantity

Bioelectrical impedance analysis (BIA) (RD-545; Tanita Corporation, Tokyo, Japan) was used for body composition measurements. This device measures body composition from four extremities (right hand, left hand, right foot, left foot) with dual frequency (50 kHz and 6.25 kHz) technology and obtains acceptable measurements [15]. BIA measurements were performed by a well-trained investigator. Measurements were taken in the morning, at least 8 h after the last meal. During the measurement, the patients’ metal accessories (such as watches, necklaces), shoes and heavy clothing were removed and the room temperature was at 20–25 °C during the measurement. Fat-free mass (FFM) was assessed via bioelectrical impedance analysis, and SMM was derived using the equation: SMM (kg) = 0.566 × FFM. This equation has been validated with both individual and group data as reported in the literature [16]. Since muscle mass quantity varies with body size, SMM was indexed according to height square (SMM-I(height^2^)) and BMI (SMM-I(BMI)).

### Physical performance

In our study, “Timed-Up and Go” test (TUG) was used to evaluate muscle function. The test was used to determine the presence of severe sarcopenia in patients diagnosed with confirmed sarcopenia. Before starting the test, patients were taught how to perform the test with a short video. The time taken to complete the test, which consist of standing up from a standard chair, walking to a marker 3 meters away, walking back and sitting down, was recorded in seconds [17].

### Muscle quality

Muscle quality is a relatively new term and muscle function to delivered per unit of muscle mass is a sign of muscle quality [11]. In our study, dividing HGS by SMM was used to evaluate muscle quality (HGS/SMM).

### Sarcopenia diagnosis

**Fig. 2:**
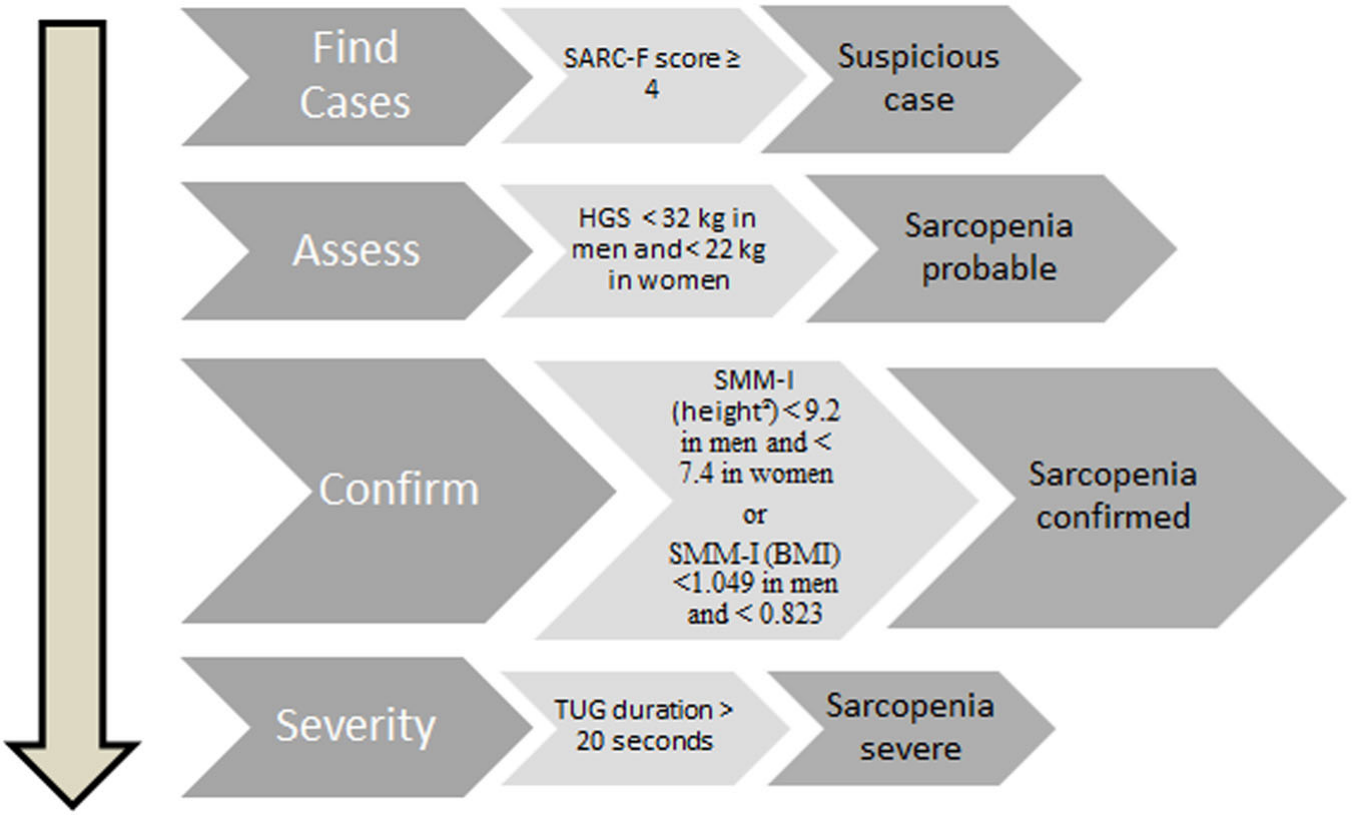
Sarcopenia diagnosis flow chart * HGS hand grip strength, SMM-I (height2) skeletal mass index adjusted by height square, SMM-I (BMI) skeletal mass index adjusted by BMI, TUG “Timed-Up and Go” test

To diagnose sarcopenia, EWGSOP2 guidelines and population specific values were taken into account. “Find-Assess-Confirm-Severity” (F-A-C-S) steps were followed in making the diagnosis, as recommended in the consensus (Fig. 2). SARC-F score ≥4 was accepted for suspicious case identification, HGS test values of <32 kg in men and <22 kg in women were accepted for the diagnosis of weak muscle strength, and TUG duration >20 s were accepted for low physical performance. For low muscle mass (LMM) confirmation, skeletal muscle mass indexed according to two different variables was evaluated. For SMM-I(height^2^), the following cut-off values were used: <9.2 kg/m^2^ in men, <7.4 kg/m^2^ in women; and for SMM-I(BMI), <1.049 kg/BMI in men / < 0.823 kg/BMI in women. EWGSOP2 recommended some specific cut-offs, but also recommended to use the normative data of the population when available. Cut-offs for total skeletal muscle mass (indexed for height^2^ and BMI) and handgrip strength have been previously calculated for Turkish population. Therefore, it was preferred to use population specific values for these parameters [11, 18–20]. At the end of these evaluations, patients are diagnosed as non-sarcopenic, sarcopenia probable, sarcopenia confirmed and sarcopenia severe [11].

### Statistical analysis

The concordance of the quantitative data to normal distribution was examined using the Kolmogorov-Smirnov test. In the comparison of normally distributed variables between groups, One-Way ANOVA test was used and descriptive statistics were shown as mean ± standard deviation. In the intergroup comparisons of non-normally distributed variables, Kruskal-Wallis test was used and descriptive statistics were given as median (25–75 percentile). In the analysis of quantitative variables, bivariate relationships were evaluated using Spearman’s correlation and presented graphically. Categorical variables were assessed using chi-square analysis and the results were presented in the form of frequency (%). Results were considered statistically significant when p < 0.05.

## Results

The demographic data and sarcopenia parameters of the study groups (NFAI = 63, MACS = 31) and the control group (n = 44) are listed in Table 1. In the NFAI group, the average age was 54.32 ± 10.64 years and 77.8% of the group was female (49 female and 14 male). In the MACS group, the average age was 57.48 ± 8.22 years and 77.4% of the group was female (24 female and 7 male). In the control group, the average age was 53.77 ± 9.18 years and 70.5% of the group was female (31 female and 13 male). There was no statistical difference between the groups in terms of age, gender, BMI, smoking status and alcohol use. While SMM-I(height^2^) did not differ between groups; HGS, HGS/SMM, total muscle mass ratio (total body), skeletal muscle mass ratio (total body) and SMM-I(BMI) was the lowest in the MACS group and the second-lowest in the NFAI group, and the results were statistically significant in both groups compared to the control group. Fat ratio (total body) was the highest in the SC group, and was second-highest in the NFAI group, and the results were statistically significant in both groups compared to the control group.

**Table 1:** Comparison of demographic data and sarcopenia parameters of all cases

	NFAI (n = 63)	MACS (n = 31)	Control group (n = 44)	*p* value
Age^a^	54.32 ± 10.64	57.48 ± 8.22	53.77 ± 9.18	0.224
Gender (Female %)^c^	77.8	77.4	70.5	0.657
BMI (kg/m^2^)^b^	31.2 (27.1–34.4)	29.4 (27.1–33.5)	29.2 (26–33.2)	0.217
FFM (kg)^b^	47.5 (40.6–52.6)	47.5 (42.8–54.5)	49.8 (45.9–58.9)	0.074
SMM (kg)^b^	26.88 (24.20–30.86)	26.88 (22.99–29.76)	28.20 (25.46–33.34)	0.074
SMM-I (height^2^)^b^	10.66 (9.85–11.48)	10.36 (9.87–11.01)	10.60 (10.09–11.68)	0.326
SMM-I (BMI)^a^	0.911 ± 0.211	0.891 ± 0.189	1.01 ± 0.194	0.012*
Fat mass (%)^a^	38.8 ± 8.4	39.2 ± 9.1	34.4 ± 8.0	0.013*
Total muscle ratio(%)^a^	58.0 ± 8.0	57.7 ± 8.6	62.3 ± 7.6	0.013*
Skeletal muscle ratio^a^ (SMM/ body weight)	34.6 ± 4.8	34.4 ± 5.1	37.2 ± 4.6	0.010*
HGS (kg)^b^	27.0 (23.0–31.0)	25.0 (22.0–31.0)	30.0 (26.0–37.25)	0.004*
Skeletal muscle quality (HGS/SMM)^a^	1.002 ± 0.191	1.020 ± 0.186	1.098 ± 0.186	0.034*
Calf circumference (cm)^b^	36 (33–39)	34 (33–39)	35 (32–38)	0.495
Mid-arm circumference (cm)^a^	29.3 ± 2.6	29.7 ± 3.4	29.6 ± 3.9	0.827

*NFAI* non-functioning adrenal incidentaloma, *MACS* mild autonomic cortisol secretion, *BMI* body mass index, *FFM* free fat mass, *SMM* skeletal muscle mass, *SMM-I (height*^*2*^*)* skeletal mass index adjusted by height square, *SMM-I (BMI)* skeletal mass index adjusted by BMI, *HGS* hand grip strength

*p < 0.05 is considered significant

^a^One-way analysis of variance (ANOVA) [mean ± standard deviation]

^b^Kruskal-Wallis Test [median (%25-75 percentile)]

^c^Chi-square test

Since it is known that muscle mass varies significantly according to gender, after the whole group analysis, the data were evaluated by subgroup analyzes for men and women separately. Additionally, due to the high number of postmenopausal individuals in the female patient group, to eliminate potential hormonal effects, analyses were conducted separately for the postmenopausal female subgroup. Comparison of demographic data and sarcopenia parameters of female cases between groups is given in Table 2, comparison of demographic data and sarcopenia parameters of postmenopausal cases between groups Table 3 and comparison of demographic data and sarcopenia parameters of male cases between groups is given in Table 4. In female and postmenopausal subgroup analyses, there was no significant difference between the groups in terms of age, BMI, smoking status and alcohol use. In the female subgroup analysis HGS, HGS/SMM, skeletal muscle mass ratio (total body) and SMM-I (BMI) maintained statistically significant differences (Table 2). In the postmenopausal subgroup analysis HGS, HGS/SMM, and SMM-I (BMI) maintained statistically significant differences (Table 3). This difference did not persist in male subgroup analyses (Table 4).

**Table 2:** Comparison of demographic data and sarcopenia parameters of female cases between groups

	NFAI (n = 49)	MACS (n = 24)	Control group (n = 31)	*p* value
Age^a^	53.98 ± 10.50	56.79 ± 7.35	53.16 ± 9.50	0.352
BMI (kg/m^2^)^b^	30.4 (26.9–34.4)	31.5 (27.4–34.3)	30.0 (25.8–33.3)	0.493
Menopause, n (%)^c^	77.6	83.3	80.6	0.836
FFM (kg)^a^	45.17 ± 5.49	45.42 ± 4.83	48.13 ± 7.58	0.093
SMM (kg)^b^	26.88 (24.20–30.86)	26.88 (22.99–29.76)	28.20 (25.46–33.34)	0.161
SMM-I (height^2^)^a^	10.39 ± 0.84	10.14 ± 0.81	10.41 ± 0.95	0.445
SMM-I (BMI)^a^	0.841 ± 0.170	0.826 ± 0.162	0.923 ± 0.127	0.037*
Fat mass (%)^a^	40.7 ± 8.1	42.4 ± 7.7	37.5 ± 6.8	0.053
Skeletal muscle ratio (SMM/ body weight)^a^	33.5 ± 4.5	32.6 ± 4.3	35.5 ± 4.0	0.043*
HGS (kg)^b^	24.0 (21.0–28.0)	24.0 (22.0–26.0)	27.0 (25.0–32.0)	0.002*
Skeletal muscle quality (HGS/SMM)^a^	0.967 ± 0.173	0.967 ± 0.145	1.059 ± 0.171	0.039*
Calf circumference (cm)^a^	36.5 ± 5.3	36.0 ± 4.0	35.4 ± 4.0	0.578
Mid-arm circumference (cm)^a^	29.5 ± 3.3	29.7 ± 2.6	29.6 ± 4.3	0.956

*NFAI* non-functioning adrenal incidentaloma, *MACS* mild autonomic cortisol secretion, *BMI* body mass index, *FFM* free fat mass, *SMM* skeletal muscle mass, *SMM-I (height*^*2*^*)* skeletal mass index adjusted by height square, *SMM-I (BMI)* skeletal mass index adjusted by BMI, *HGS* hand grip strength

*p < 0.05 is considered significant

^a^One-way analysis of variance (ANOVA) [mean ± standard deviation]

^b^Kruskal-Wallis Test [median (%25–75 percentile)]

^c^Chi-square test

**Table 3:** Comparison of demographic data and sarcopenia parameters of postmenopausal cases between groups

	NFAI (n = 38)	MACS (n = 20)	Control group (n = 25)	*p* value
Age^a^	54 (52–63)	59 (54.25–62.75)	55 (50.5–62)	0.339
BMI (kg/m^2^)^b^	32.1 (27.85–36.62)	31.5 (27.1–34.77)	30.3 (25.35–33.6)	0.469
FFM (kg)^a^	45.32 ± 5.19	44.02 ± 4.22	47.56 ± 7.61	0.121
SMM (kg)^b^	25.65 ± 2.93	24.91 ± 2.39	26.92 ± 4.31	0.121
SMM-I (height^2^)^a^	10.5 ± 0.86	10.04 ± 0.55	10.31 ± 0.96	0.148
SMM-I (BMI)^a^	0.822 ± 0.172	0.809 ± 0.164	0.912 ± 0.126	0.049*
Fat mass (%)^a^	41.43 ± 7.86	42.72 ± 8.01	38.02 ± 6.58	0.091
Skeletal muscle ratio (SMM/ body weight)^a^	33.14 ± 4.43	32.42 ± 4.52	35.07 ± 3.73	0.092
HGS (kg)^b^	23.92 ± 4.41	24.1 ± 3.68	28.28 ± 5.24	0.001*
Skeletal muscle quality (HGS/SMM)^a^	0.936 ± 0.161	0.972 ± 0.148	1.058 ± 0.160	0.014*
Calf circumference (cm)^a^	35 (32–38)	35.5 (33.25–40.75)	35 (32–39)	0.906
Mid-arm circumference (cm)^a^	30 (27–32.25)	30 (27–32)	30 (25–32)	0.974

*NFAI* non-functioning adrenal incidentaloma, *MACS* mild autonomic cortisol secretion, *BMI* body mass index, *FFM* free fat mass, *SMM* skeletal muscle mass, *SMM-I (height*^*2*^*)* skeletal mass index adjusted by height square, *SMM-I (BMI)* skeletal mass index adjusted by BMI, *HGS* hand grip strength

*p < 0.05 is considered significant

^a^One-way analysis of variance (ANOVA) [mean ± standard deviation]

^b^Kruskal-Wallis Test [median (%25-75 percentile)]

**Table 4:** Comparison of demographic data and sarcopenia parameters of male cases between groups

	NFAI (n = 14)	MACS (n = 7)	Control group (n = 13)	*p* value
Age^a^	56.5 (46.25–62.75)	59 (57–70)	59 (50–63)	0.546
BMI (kg/m^2^)^b^	31.6 (28.4–35.4)	28.4 (26.2–29.1)	29.1 (26.0–30.8)	0.046*
FFM (kg)^a^	63.32 ± 8.02	54.49 ± 3.84	61.75 ± 7.85	0.042*
SMM (kg)^b^	35.22 (32.41–39.38)	30.94 (29.76–31.63	34.87 (30.66–39.10)	0.023*
SMM-I (height^2^)^a^	11.78 (11.08–13.09)	11.34 (10.85–11.47)	11.88 (10.80–12.48)	0.224
SMM-I (BMI)^a^	1.152 (1.029–1.220)	1.094 (1.046–1.182)	1.199 (1.139–1.350)	0.085
Fat mass (%)^a^	33.6 (31.0–36.6)	28.6 (26.0–31.7)	28.4 (22–29.9)	0.005*
Skeletal muscle ratio (SMM/ body weight)^a^	37.6 (35.9–39.0)	40.2 (38.7–41.8)	40.5 (39.7–41.8)	0.005*
HGS (kg)^b^	39 (32–48.2)	38 (30–42)	42 (33–48.5)	0.466
Skeletal muscle quality (HGS/SMM)^a^	1.114 (0.978–1.280)	1.208 (0.969–1.411)	1.225 (1.001–1.354)	0.561
Calf circumference (cm)^a^	35 (33–38.5)	34 (34–36)	35.5 (33.75–40)	0.677
Mid-arm circumference (cm)^a^	30.5 (29–31)	28 (26–29)	30 (28.5–31.5)	0.063

*NFAI* non-functioning adrenal incidentaloma, *MACS* mild autonomic cortisol secretion, *BMI* body mass index, *FFM* free fat mass, *SMM* skeletal muscle mass, *SMM-I (height*^*2*^*)* skeletal mass index adjusted by height square, *SMM-I (BMI)* skeletal mass index adjusted by BMI, *HGS* hand grip strength

*p < 0.05 is considered significant

^a^One-way analysis of variance (ANOVA) [mean ± standard deviation]

^b^Kruskal-Wallis Test [median (%25–75 percentile)]

**Fig. 3:**
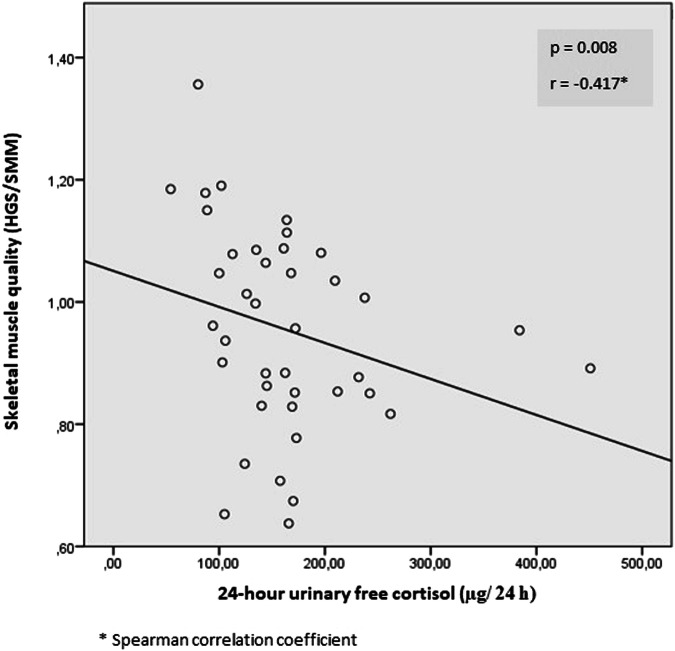
Correlation analysis of skeletal muscle quality and 24-h urinary free cortisol in female patients * HGS hand grip strength, SMM skeletal mass

**Fig. 4:**
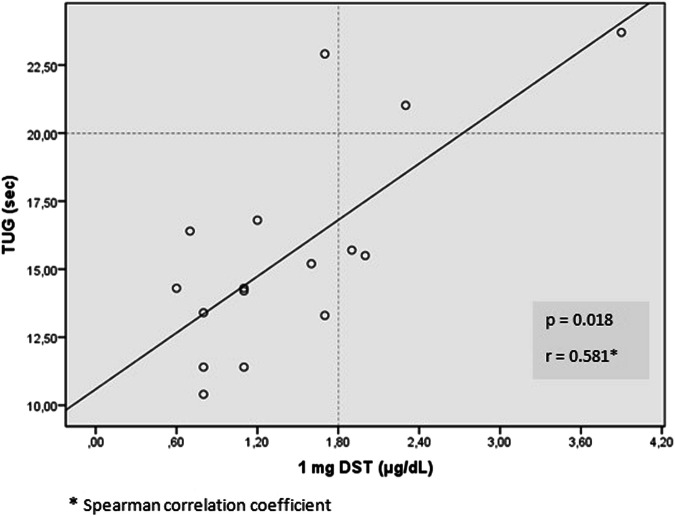
Correlation analysis of TUG and post-1 mg DST cortisol in patients with confirmed sarcopenia * TUG “Timed-Up and Go” test, SMM skeletal mass, DST dexamethasone suppression test

In the correlation analysis of the women subgroup, it was seen that the 24-h free urine cortisol value was inversely proportional to the muscle quality (HGS/SMM) (Fig. 3). In addition, it was observed that TUG values of confirmed sarcopenia patients showed a positive correlation with post-1 mg DST cortisol (Fig. 4).

When SMM-I (BMI) was used to detect LMM, confirmed sarcopenia was detected at a rate of 19.15% (18 of 94 patients) and severe sarcopenia was detected in 3.2% of the patients (3 of 94) in AI group (63 NFAI and 31 MACS). When SMM-I (height^2^) was used to detect LMM, confirmed sarcopenia was detected at a rate of 5.32% (5 of 94 patients) and severe sarcopenia was detected in 2.13% of the patients (2 of 94) in AI group (63 NFAI and 31 MACS). The results did not show a statistically significant difference between the groups. When EWGSOP2 cut-offs ( < 27 kg for males and <16 kg for females) were used for HGS rather than population-specific values, it was observed that only 2.13% of the patients (2 of 94) were diagnosed with confirmed sarcopenia. These two patients were also diagnosed as severe sarcopenia with their TUG values.

## Discussion

The main finding of our study states that muscle strength, muscle mass, muscle quality and muscle performance decreases in AI patients, and this decrease is more pronounced in MACS group. The negative correlation between muscle quality and 24-h urinary free cortisol in MACS patients suggests the role of hypercortisolism in the pathogenesis. The extension at the duration of TUG as the post-1 mg DST cortisol level increases in patients with confirmed sarcopenia can be considered as another finding supporting this. When SMM-I (BMI) is used to indicate LMM, the frequency of sarcopenia is higher. Thus, this parameter is more sensitive in diagnosing sarcopenia compared to SMM-I (height^2^). These two parameters have also been evaluated in other studies for their relationship with sarcopenia parameters. The EWGSOP2 authors did not favor either parameter and stated that population specific values could be used if available. FNIH has recommended the use of SMM-I (BMI) and current studies favor SMM-I (BMI) rather than SMM-I (height^2^) [21–23].

The number of studies evaluating AI & MACS-related sarcopenia in literature is limited. Delivanis DA et al. compared CS, MACS, and NFAI patients with abdominal CT for muscle mass and visceral adiposity in their study. They found out that hypercortisolism (morning post-1 mg DST cortisol) was positively correlated with visceral adiposity and negatively correlated with muscle mass [6]. Similar to our study, Kim JH and colleagues compared patients with subclinical hypercortisolism and NFAI by separating them into two groups as male and female in terms of muscle mass by BIA. In this study, skeletal muscle mass was found to be lower in the female subclinical hypercortisolism group than the NFAI group [24]. In these two studies, it was discussed that decreased muscle mass and increased fat percentage in MACS and CS patients may be due to increased activity of 11-beta-hydroxyreductase (11β-HSD1) activity. This may be due to 11β-HSD1 increasing the glucocorticoid effect in muscle cells by affecting the intracrine and paracrine control of glucocorticoid signaling [25].

In our study, the number of female patients was higher in the AI and SC groups. Different rates are given in the literature regarding the distribution of AI by gender, but it is known that AI is more common in female. A working group on adrenal tumors of the Italian Society of Endocrinology reported that AIs occur more frequently in women (58.0%, 584 out of 1004) [26]. A population-based cohort study that evaluated adrenal tumors epidemiologically in the USA also reported that adrenal tumors were more common in women. In this study, the distribution of patients with adrenal tumors by gender was found to be higher in women with 55.4%. After characterization of the tumors, adrenocortical adenoma and nodular hyperplasia were found to be more common in women with 57.1% [27]. The high percentage of women in our control group, like our study group, contributed to the reliability of our statistical results.

The present study also provided data on the incidence of confirmed sarcopenia and severe sarcopenia in AI patients for the first time. The frequency of confirmed sarcopenia was 19.15% (18 of 94 patients), the frequency of severe sarcopenia was 3.2% (3 of 94 patients). Although there are different data in the literature regarding the frequency of sarcopenia, in general, sarcopenia affects 10–16% of the elderly worldwide [28]. Pang BWJ and colleagues evaluated 542 patients (57.9% women) aged 21-90 years for the presence of sarcopenia. In this study, based on EWGSOP2 criteria, the prevalence of confirmed sarcopenia was found to be 1.3% and the prevalence of severe sarcopenia was also found to be 1.3% between the ages of 21–60. In patients aged 60 and above, the prevalence of confirmed sarcopenia was 7.3% and the prevalence of severe sarcopenia was 16.8% [29]. Considering that the mean age in the AI patient group in our study was found to be 56.5 ± 8.22, it can be concluded that the frequency of sarcopenia increases with age in AI.

There is still debate in the literature whether to use population-specific cut-off values or consensus-recommended fixed cut-off values for the diagnosis of sarcopenia. EWGSOP2 provides recommendations on cut-off points for different parameters to improve the agreement of sarcopenia studies, while at the same time recommending the use of normative population references whenever possible, and that cut-off points are usually set as −2 standard deviation of healthy young adult values [11]. The significantly lower levels of sarcopenia detected when using EGWSOP2 cut-offs for HGS than previously reported in the literature (2.13% of patients (2 of 94)) suggests that it may be more appropriate to use population-specific cut-offs. In a study conducted in Italy, another Mediterranean country, the cut-off values for HGS were determined to be similar to our reference study (30 kg for male and 22 kg for female) [30].

In our study, loss of muscle mass and function secondary to moderate hypercortisolemia showed sexual dimorphism. Menopause may also affect muscle mass and functions in female patients due to withdrawal of estrogen. However, in our study, there was no difference in the number of postmenopausal women among three groups. Furthermore, analyses of the postmenopausal subgroup revealed that muscle quality, HGS, and SMM-I (BMI) were significantly lower in the MACS and NFAI groups compared to the control group. Similar sexual dimorphism was also observed in the study of Kim JH et al, and the author reported that this may be related to the fact that upregulation of skeletal muscle 11β-HSD1 was observed in older women, but not in men [23, 31]. In another study examining the plasma cortisol circadian rhythm of aging and gender, E Van Cauter and colleagues found that the inter-individual variability of the morning cortisol peak was greater in men than in women [32]. This difference may have caused heterogeneity in the male group. The small sample size in the male group in our study may have caused heterogeneity in the statistical analyses of this group. Table 4 shows a significant difference in BMI in the male subgroup analyses. Considering the effect of BMI on muscle mass and functions, this may be another negative factor affecting the analyses of this group. Testosterone has a potent anabolic effect on muscle mass by activating stem cells, satellite cells, myocytes, and fibroblasts by promoting protein synthesis and muscular regeneration. This protective effect of testosterone in male patients may be another reason for the absence of a significant difference between groups in male patients [33].

### Limitations and strengths

Unlike other studies, our study also compared AI and MACS patients with a healthy control group (without adrenal mass). Our study was designed with reference to EGSWOP2, one of the most current and valid guidelines on sarcopenia and in addition to measuring muscle mass, we also measured muscle strength, performance and quality. Muscle strength measurement (HGS), which comes before muscle mass in the sarcopenia algorithm, was low in MACS and NFAI patients.

Our study has several limitations. Firstly, our number of control group patients was low compared to our number of cases. However, since AI is also common in the normal population, we could not increase the number of control patients because we could only include cases with CT or MRI scans in our control group. Another limitation of our study is that the EWGSOP2 consensus does not accept BIA as the gold standard for muscle mass measurement due to its limitations. The consensus recommends using BIA for clinical rather than research purposes. However, BIA has been compared with other methods in many studies in calculating SMM and the results have been found to be correlated [34, 35]. Moreover, it can be applied more easily in clinical practice than other muscle mass estimation and determination techniques (DXA, CT, MRI), is much more effective and does not require irradiation.

## Conclusion

Our study showed that there is a decrease in muscle mass and function in female AI patients, and this decrease more severe in MACS patients. These results may suggest that mild cortisol excess also has negative effects on skeletal muscle metabolism. MACS patients and even NFAI patients (especially females) should also be evaluated with muscle mass and function measurements to determine the risk of sarcopenia and prevent possible complications.

## Data Availability

No datasets were generated or analysed during the current study.
